# 

**Published:** 1837-07

**Authors:** 


### CONTENTS OF No. VII.

OF THE

### British and Foreign Medical Review.

JULY, 1837.

---

### Part First.—Analytical and Critical Reviews.

PAGE

Art. I.—1. An Introduction to Botany. By John Lindley, ph. d., f.r.s., l.s. &c. 1
2. Library of Useful Knowledge. Botany. . . . . . ib.
3. The Principles of Descriptive and Physiological Botany. By the Rev. J. S. Henslow, m.a. f.l.s., &c. . . . . . ib.
4. Introduction à l'Etude de la Botanique, &c. Par M. Alph. De Candolle . ib.
Introduction to the Study of Botany, &c. By M. Alph. De Candolle.
5. Physiologie der Gewächse. Von Ludolph Christian Treviranus, m.d. &c. ib.
Vegetable Physiology. By L. C. Treviranus, m.d. and ph.d.
6. Nouveau Système de Physiologie Végétale et de Botanique, &c. Par F. V. Raspail . . . . . . . ib.
New System of Vegetable Physiology and Botany, &c. By F. V. Raspail.

Art. II.—Lectures on the Morbid Anatomy of the Serous and Mucous Membranes. By Thomas Hodgkin, m.d. &c. . . . . 31

Art. III.—1. Observations on the Influence of Religion upon the Health and Physical Welfare of Mankind. By Amariah Brigham, m.d. . . . 55
2. Remarks on the Influence of Mental Cultivation and Mental Excitement upon Health. By Amariah Brigham, m.d. . . . . ib.

Art. IV.—De la Prostitution dans la Ville de Paris, considérée sous la Rapport de l'Hygiène publique, de la Morale, et de l'Administration; ouvrage appuyé de Documens statistiques puisés dans les Archives de la Préfecture de Police. Par A. J. B. Parent-Duchatelet, &c. . . . . 63
On Prostitution in the City of Paris, &c. By A. J. B. Parent-Duchatelet.

Art. V.—The Works of John Hunter, f.r.s.; with Notes. Edited by James F. Palmer, Senior Surgeon to the St. George's and St. James's Dispensary, &c. 75

Art. VI.—1. The Proofs of Infanticide considered. By Wm. Cummin, m.d. . 87
2. Beiträge zur Lehre von dem Thatbestande des Kindermordes überhaupt, und den ungewissen Todesarten neugeborner Kinder insbesondere. Von Dr. Schwörer ib.
Contributions to the Doctrine of Infanticide, more especially in Relation to the doubtful Causes of Death in New-born Children. By Dr. Schwörer.
3. Researches in Medicine and Medical Jurisprudence. By John B. Beck, m.d. ib.

Art. VII.—1. Pharmacopœia Collegii Regalis Medicorum Londinensis . 101
2. The Translation of the Pharmacopœia of the Royal College of Physicians of London, 1836. With Notes and Illustrations. By R. Phillips, f.r.s. l.&e. ib.
3. The Pharmacopœia Collegii Regalis Medicorum Londinensis, translated, with a Commentary, chemical, pharmaceutical, and medicinal. By D. Spillan, m.d. ib.
4. A Translation of the New Pharmacopœia of the Royal College of Physicians of London; with Notes and Criticisms. By G. F. Collier, m.d. . . ib.

Art. VIII.—System der Physiatrik, oder der Hippokratischen Medicin. Von Ferdinand Jahn. Erster Band. Physiologie der Krankheit und des Heilungsprocesses, oder allgemeine Pathologie und Jatreusiologie . 120
A System of Physiatrics, or of Hippocratic Medicine. By F. Jahn. First Vol. Physiology of Disease and Restoration, or General Pathology and Iatreusiology.

Art. IX.—Lectures illustrative of certain Local Nervous Affections. By Sir Benj. C. Brodie, Bart., f.r.s., &c. . . . . . 132

PAGE

ART. X.—Medico-Chirurgical Transactions, published by the Royal Medical and Chirurgical Society of London, for 1836. Vol. XX. . . . 138
Dr. YELLOLY on Vascular Appearances of Mucous and Serous Membranes, &c. ib.
Mr. J. G. PERRY's Remarkable Case of Varicose Aneurism, with Observations ib.
Mr. SAMPSON's Case of Recovery from the Insensibility of Intoxication, &c. 140
Mr. STAFFORD on the Treatment of Injuries received in Dissection . ib.
Mr. PHILLIPS' Case of Fracture and Displacement of the Atlas . . 141
Mr. SMITH on artificial Respiration in some Cases of Poisoning with Opium . 142
Dr. KINGSTON's Remarks on two Forms of Atrophy of the Heart's Valves, &c. ib.
Mr. BENJAMIN TRAVERS', Jun., Case of unusually dislocated Thigh-bone 143
Sir B. C. BRODIE on Injuries of the Spinal Cord . . . ib.
Mr. LISTON's Observations on some Tumours of the Mouth and Jaws . 147
Dr. BURNE on Inflammation, Chronic Disease, and perforative Ulceration of the Cæcum . . . . . . . 148
Dr. W. THOMSON on black Expectoration and black Matter in the Lungs . 150
Mr. NORMAN's Case of Ligature of the external Iliac Artery . . 151
Dr. KINGSTON on some Points of the Pathology of Pulmonary Tubercles . ib.
Mr. CURLING's Observations on some of the Forms of Atrophy of Bone . 152

ART. XI.—Ueber die anwendung der Ligatur an einer von der Wunde entfernten, dem herzen zugewendeten stelle der verwundeten Arterie oder des entsprechenden Arterienstammes. Ein Beitrag zur Therapie der traumatischen Blutungen von KARL JOSEPH BECK, M.D. &c. . . . 154
On the Application of the Ligature to Wounded Arteries, or their Trunks, at a Distance from the wounded Part and nearer the Heart. An Essay on the Treatment of Traumatic Hemorrhages. By K. J. BECK, M.D.

ART. XII.—A Clinical Treatise on the Endemic Fevers of the West Indies, intended as a Guide for the young Practitioner in those Countries. By Mr. EVANS 160

ART. XIII.—Chirurgische Kupfertafeln, zum Gebrauch für practische Chirurgen. Herausgegeben von Dr. ROBERT FRORIEP . . . . 168
Surgical Copper-plates, for the Use of Practical Surgeons. Published by Dr. ROBERT FRORIEP.

ART. XIV.—1. A Practical Treatise on the Diseases of the Skin, arranged with a View to their Constitutional Causes and local Characters, &c. By Mr. PLUMBE 175
2. A Practical Compendium of the Diseases of the Skin, including a particular Consideration of the more frequent and intractable Forms of these Affections; with Cases. By JONATHAN GREEN, M.D. &c. . . . . ib.

ART. XV.—1. Practical Observations on various Subjects relating to Midwifery. By JAMES HAMILTON, M.D. F.R.S.E. &c. . . . . 181
2. A Letter addressed to Dr. FORBES and Dr. CONOLLY, Editors of the British and Foreign Medical Review. By Dr. HAMILTON, Professor of Midwifery, &c. ib.

ART. XVI.—First Principles of Surgery; being an Outline of Inflammation and its Effects. By GEORGE T. MORGAN, A.M. . . . . 185

---

### PART SECOND.—Bibliographical Notices.

ART. I.—The Hunterian Oration, delivered in the Theatre of the Royal College of Surgeons in London, on the 14th of February, 1837. By Sir BENJAMIN C. BRODIE, Bart. F.R.S. &c. . . . . . 189

ART. II.—A Letter to the Right Honorable Sir HENRY HARDINGE, K.C.B. M.P. on the Effects of Solitary Confinement on the Health of Soldiers, in Warm Climates. By JOHN GRANT MALCOLMSON, F.R.A.S. and M.G.S. . . 191

ART. III.—1. An Introductory Lecture, delivered in the Anatomical Theatre of the University of Maryland, on the 1st November, 1836. By Dr. GRIFFITH . 193
2. An Inaugural Address on Medical Eclectism. By J. CONQUEST CROSS, M.D. ib.

ART. IV.—The Cyclopædia of Practical Surgery, comprising a Series of original Dissertations on Operative Medicine, by an Association of Physicians and Surgeons. Edited by W. B. COSTELLO, M.D. . . . 195

ART. V.—Medical Essays. By J. HUNGERFORD SEALY, M.D. E.A.B. T.C.D. No. I. Phthisis Pulmonalis, curable and incurable. No. II. The Imagination, its history and effects . . . . . . . 196

PAGE

ART. VI.—An Address delivered to the Members of the Worcestershire Natural History Society, on the Opening of the Worcestershire Museum, September 15, 1836. By CHARLES HASTINGS, M.D. F.G.S. . . . 197

ART. VII.—Dissertationis de Signis Mortem hominis absolutam ante Putredinis accessum indicantibus particula prior et posterior. Auctore A. G. SOMMER, M.D. ib.
Dissertation on the Signs of certain Death, previously to Putrefaction. By A. G. SOMMER, M.D.

ART. VIII.—A Dictionary of Practical Medicine; comprising general Pathology, the Nature and Treatment of Diseases, &c. By JAMES COPLAND, M.D. &C. 200

---

### PART THIRD.—Selections from Foreign Journals.

### ANATOMY AND PHYSIOLOGY.

On Artificial Digestion. By Professor MÜLLER and Dr. SCHWANN . . 201
Observations on Weber's Experiment on the Power by which the Head of the Thigh Bone is retained in the Acetabulum. By Dr. LAUER . . 205
A single Artery for the two Ventricles of the Heart, Cyanosis . . 207
On the Action of the Acetate of Lead upon the Animal Economy. By Professor C. G. MITSCHERLICH, M.D. . . . . . 208
On the Origin of the Fifth and Seventh Pair of Nerves. By A. RETZIUS . 212

### PATHOLOGY, PRACTICAL MEDICINE, AND THERAPEUTICS.

On the Indications for the Use of Chlorine and Muriatic Acid Vapours in Diseases of the Air-Passages and Lungs. By Professor ALBERS, of Bonn . ib.
Professors SEBASTIAN and ALBERS on Tubercles . . . . 216
On the Indications for the Use of Moxa, with Cases. By Dr. SADLER . 217
Contest between Scarlatina and Small-pox. By DR. GLEHN . . 219
On the Treatment of Ileus with Belladonna Clysters. By Dr. WAGNER . 220
Remarkable Case of Pica. By Dr. VON VOGEL, of Rostock . . 222
Rare Case of Spontaneous Human Combustion. By Dr. PATRIX . . ib.
Tobacco in Tetanus. By Dr. CAVENUE, of Martinique . . 223
Observations on the Vaccine Pustule. By M. DUCROS, Jun. . . ib.
Use of Chlorine Inhalations in acute and chronic Bronchitis, and in Bronchorrhœa. By M. A. TOULMOUCHE, of Rennes . . . . 224

### SURGERY.

Observations on Hypertrophy of the Mammary Gland. By Dr. FINGERHUTH . ib.
On the Forms of Lepra observed in the Russian Provinces bordering on the Baltic. By Dr. BLOSFELD, of Riga . . . . . 227
On Excision of the smaller Joints. By Dr. GERNET, of Hamburg . . 230
On the Treatment of Varus and Pes Equinus, by the division of the Tendo Achillis. By Dr. BOUVIER . . . . . . 231
Account of a new Instrument for plugging the posterior Nares. By M. SAINT ANGE ib.
Treatment of Sprains by Friction and Shampooing. By Dr. MAIGNIEN . 232
Make-shift Surgery. By Dr. NEVERMANN, of Mecklenburg-Schwerin . ib.
On the Treatment of Varicocele. By Dr. FRICKE, of Hamburg . . ib.

### MIDWIFERY.

Fracture of the Skull and Rupture of the longitudinal Sinus during Natural Labour. By Dr. MICHAELIS, of Kiel . . . . . 233

### MEDICAL STATISTICS.

Statistics of Suicide . . . . . . . 234
Statistics of Stillborn Children . . . . . . ib.

### TOXICOLOGY.

Experiments on the Effects of the Hydrated Per-Oxyde of Iron as an Antidote to Arsenious Acid. By Dr. VON SPECZ . . . . 237
Case of Poisoning by Liquor Potassæ. By DARTO-MASSART . . 239

### VETERINARY SURGERY.

Experimental Researches regarding Hydrophobia. By M. CAPELLO . . 240

### Part Fourth.—Selections from British Journals.

ANATOMY AND PHYSIOLOGY. PAGE

A Description of the Pulmonic Pulse. By P. Mollison, m.d. . . 241

Physiological Observations on the Pulsations of the Heart. By Dr. Knox . ib.

PATHOLOGY, PRACTICAL MEDICINE, AND THERAPEUTICS.

On the External Application of Opium in Bronchitis and Croup. By Dr. Bow . 243
On Castor-Oil Frictions in Gout. By W. E. Pope, Esq. . . . ib.
On Nux Vomica in Affections of the Digestive Organs. By Thomas Mellor, Esq. 244
An Account of Tubercles in the Air-Cells of a Bird, and some Observations on Tubercles in general. By R. Harrison, m.d. &c. . . . ib.
On the Tonic Treatment of Erysipelas. By Henry Bullock, Esq. . . 245
On the "Bruit du Diable." By T. O. Ward, m.d. . . . ib.
Taste of Quina . . . . . . . 246
Remarks on the Treatment of Hydrocephalus Acutus. By Dr. Mayo . ib.
On the Influence of Gravity on the Circulation of the Blood. By F. R. Mosely, Esq. ib.
Clinical Report of the Cases treated in the Fever Ward, No. 9, of the Royal Infirmary, Edinburgh, during the Year 1836-7. By David Craigie, m.d. f.r.s.e. . 247
Remarks on the Physiological and Therapeutical Effects of Colchicum. By Robert Lewins, m.d. . . . . . . 249
Cure of Headach by Leeches to the Schneiderian Membrane. By J. Walker, m.d. 251
Case in which seven Half-crowns had been swallowed. By H. Wakefield, Esq. ib.
On the Treatment of various Diseases by means of Creosote. By Sir F. Smith, m.d. ib.
Medical Problems. By William Griffin, m.d., Limerick . . . 252
Diabetes cured by Diuretics. By Henry Snowden, Esq., Hull . . 254
Case of severe Cough, ending in Rupture of the Lung, &c. By F. G. Hicks, Esq. ib.
On the Use of the Nitro-muriatic Bath. By C. Lendrick, m.d. &c. . . ib.
The History of a very extraordinary and unusually violent Case, (supposed to have been engendered by Glanders,) which terminated fatally. By Mr. Brown 255

SURGERY.

Case of Popliteal Aneurism. By R. Middlemore, Esq., Birmingham . 256
Compound Luxation of the Humerus. By P. T. Scott, Esq. . . ib.
On the Operation for Hernia, without dividing the Sac. By M. W. Hilles, Esq. ib.
Cases, &c. illustrative of Subjects in Military Surgery. By Sir G. Ballingall . ib.
Observations on Extraction and Displacement of the Cataract. By Dr. Robertson 257
Application of solid Nitras Argenti in the Gonorrhœa of Women. By Dr. Hannay 258
Spontaneous Mortification of the Toes treated by tightly Bandaging the Leg. By J. C. Spender, Esq., Bath . . . . . 259
Case of Laceration of the Diaphragm. By T. B. Curling, Esq. . . 260

MIDWIFERY.

Observations on the artificial Dilatation of the Mouth of the Womb during Labour, and upon instrumental Delivery, &c. By Robert Collins, m.d. &c. . ib.
An Inquiry into the Management of the first Stage of Labour. By Dr. Murphy . ib.
On the Length of the Umbilical Cord, and its mechanical Influence upon Parturition. By F. Churchill, m.d. . . . . . 261

MEDICAL STATISTICS.

Statistics of the Negro Slave Population in the West Indies. By Mr. Tulloch . ib.
Rate of Mortality in Sweden in 1810-1830 . . . . 262

---

### Part Fifth.—Medical Intelligence.

Sketch of the present State of Medicine, and of Medical Institutions, in Russia. By George Lefevre, m.d. Part II. Of the Medical Profession and Medical Institutions . . . . . 263
Provincial Medical and Surgical Association . . . . 279
On Hepatic Abscess in India. By W. Geddes, Esq. . . . ib.
General Registration of Diseases . . . . . 280
Physiological Discoveries. By T. J. Todd, m.d. . . . 281
Extract of a Letter from Heidelberg . . . . 282
Books received for Review . . . . 283

